# Access to health and social protection policies by homeless people during the COVID-19 pandemic: a mixed-methods case study on tailored inter-sector care during a health emergency

**DOI:** 10.3389/fpubh.2024.1356652

**Published:** 2024-02-26

**Authors:** Ana Luisa Jorge Martins, Anelise Andrade de Souza, Gabriela Drummond Marques da Silva, Ana Carolina de Moraes Teixeira Vilela Dantas, Rafaela Alves Marinho, Luísa da Matta Machado Fernandes, Ana Maria Caldeira Oliveira, Helvécio Miranda Magalhães Júnior, Rômulo Paes-Sousa

**Affiliations:** ^1^Health and Social Protection Policies Research Group, René Rachou Institute/Fiocruz Minas, Oswaldo Cruz Foundation (FIOCRUZ), Belo Horizonte, Minas Gerais, Brazil; ^2^School of Nutrition, Federal University of Ouro Preto (UFOP), Ouro Preto, Minas Gerais, Brazil

**Keywords:** homeless, inter-sector collaboration, health services access, social assistance services, social protection, triangulation of methods, COVID-19

## Abstract

**Introduction:**

The article analyzed homeless people's (HP) access to health and social protection policies and tailored inter-sector care, including emergency measures, during the COVID-19 pandemic in Belo Horizonte (BH), capital of Minas Gerais state, Brazil. It intended to provide data on HP and evaluate existing public policies focused on vulnerable populations during this health emergency.

**Methods:**

The study adopted a mixed-methods design with triangulation of quantitative and qualitative data.

**Results:**

Social cartography showed that in the early months of the pandemic, the health administration had difficulty reordering the health system, which experienced constant updates in the protocols but was nevertheless consolidated over the months. The evidence collected in the study showed that important emergency interventions in the municipality of BH involved activities that facilitated access by HP to the supply of services.

**Discussion:**

The existence of national guidelines for inter-sector care for HP cannot be ruled out as a positive influence, although the municipalities are responsible for their implementation. Significantly, a health emergency was necessary to intensify the relationship between health and social protection services. Roving services were among those with the greatest positive evidence, with the least need for infrastructure to be replicated at the local level. In addition, the temporary supply of various inter-sector services, simultaneously with the provision of day shelters by organized civil society, was considered a key factor for expanding and intensifying networks of care for HP during the emergency phase. A plan exists to continue and expand this model in the future. The study concluded that understanding the inter-sector variables that impact HP contributes to better targeting of investments in interventions that work at the root causes of these issues or that increase the effectiveness of health and social protection systems.

## Introduction

Brazil experienced wide variations between state capitals in their responses to the COVID-19 health emergency. Such variations were associated with geographic inequalities and different health care capacities, highlighting the positive response by the municipality of Belo Horizonte (BH) (1), whose Greater Metropolitan Area is home to five million inhabitants, the third largest in Brazil (2). The city is known historically for strong investments in public policies, including the structuring of health systems (under Brazil's Unified Health System, known in Portuguese as *Sistema Único de Saúde*—SUS) and social protection systems (under Brazil's Unified Social Assistance System, known in Portuguese as *Sistema Único de Assistência Social*—SUAS) (3, 4). During the pandemic, public policy action in BH was known in the country for good performance in tackling COVID-19 based on strong crisis management and multidimensional responses to the emerging challenges (4). A study by Imperial College London reported that BH showed the lowest COVID-19 mortality rate among 14 Brazilian state capitals and estimated that half of the deaths in the other Brazilian cities could have been averted if they had followed the same trend as BH (1). One of the city's little-known achievements involved multiple efforts to supply care for homeless people (HP) during the pandemic, which is the object of the study reported in this article.

Homelessness puts individuals in a situation of significant social vulnerability, marked by prior living and health conditions, exposure to risk factors and violence due to limited access to financial resources, leaving them subject to constant violations of their human and social rights, and exposure to discrimination in access to health services and health care goods (5, 6). We propose that multidisciplinary approaches involving health and social protection have the potential to increase access to multiple inter-sector and specific services for HP (7, 8).

There is currently an important gap in the literature on policies for HP and responses targeted to them during health emergencies. Since HP are marked by extreme vulnerability (7, 8), this gap currently poses a clear challenge for “reaching those left behind,” a principle enshrined in the multilateral agreement on the Sustainable Development Goals (SDGs) under the 2030 Agenda for prioritization of policies targeted to more vulnerable groups (9, 10). In the early phase of the pandemic, there was a concern over the possibility of HP's increased vulnerability to COVID-19 due to their precarious status and the intersection between various chronic health conditions (11, 12). However, difficulties in obtaining public data on HP prevent the identification of the scope of this impact, while the lack of evaluation of targeted policies hinders the development of knowledge on effective responses to their specific needs. The case of BH, with its attempt to prioritize care for HP during the pandemic, thus represents an important potential contribution to the literature.

The city's positive performance is known to have benefited from Brazil's national guidelines on care for HP as a social right, by recommending targeted services and actions with continued care for this social group (rather than merely temporary interventions). Public health and social protection are Constitutional rights in Brazil, ensured as part of social welfare policy since 1988 (13, 14). The National Policy for Homeless People (known in Portuguese as *Pol*í*tica Nacional para a População em Situação de Rua*—PNPSR) was approved in 2009, drafted in dialogue with social movements and civil society for the national structuring of inter-sector policies for this population. In the operationalization of targeted policies, the PNPSR functions as a legal framework that seeks to guarantee rights and promote inter-sector assistance and comprehensive care for HP throughout the country (15).

The definition of HP in this article is the same as recommended by the PNPSR, namely, “a heterogeneous population group with the common characteristics of extreme poverty, broken or weak family ties, lack of regular conventional housing, and that uses public byways and degraded areas as spaces for temporary or permanent housing and subsistence, as well as overnight or temporary shelters as provisional housing” (15). This definition excludes people that sleep on the streets some days of the week due to pendular movement (commuting) between cities to work but who return to their homes periodically.

This article's objective was to analyze access by HP to emergency measures related to health and social protection policies and their inter-sector relations during the COVID-19 pandemic in BH until late 2021, using a mixed-methods convergent study design. The quantitative data also included 2019 as the cutoff year prior to the start of the pandemic for purposes of comparing information. The study further intends to help mitigate the gap in data on HP and in the evaluation of public policies targeted to vulnerable populations in the health emergency.

## Methods

### Study design

The article is based on a mixed-methods study with triangulation of quantitative and qualitative methods through a convergent design, also known as a parallel study or concurrent design. Considering the complexity of variables that affect HP and the difficulties in obtaining data on this population, the convergent design was adopted due to its capacity for dialogue among data of various types and complementary data on the same theme. The study's underlying paradigm is pragmatism, which features pluralist characteristics centered on the research problem and oriented by real-world practices, consequences, and limitations, as a philosophical premise that fosters openness to the aggregation of different data collection methods for studying the problem (16).

**Figure 1:**
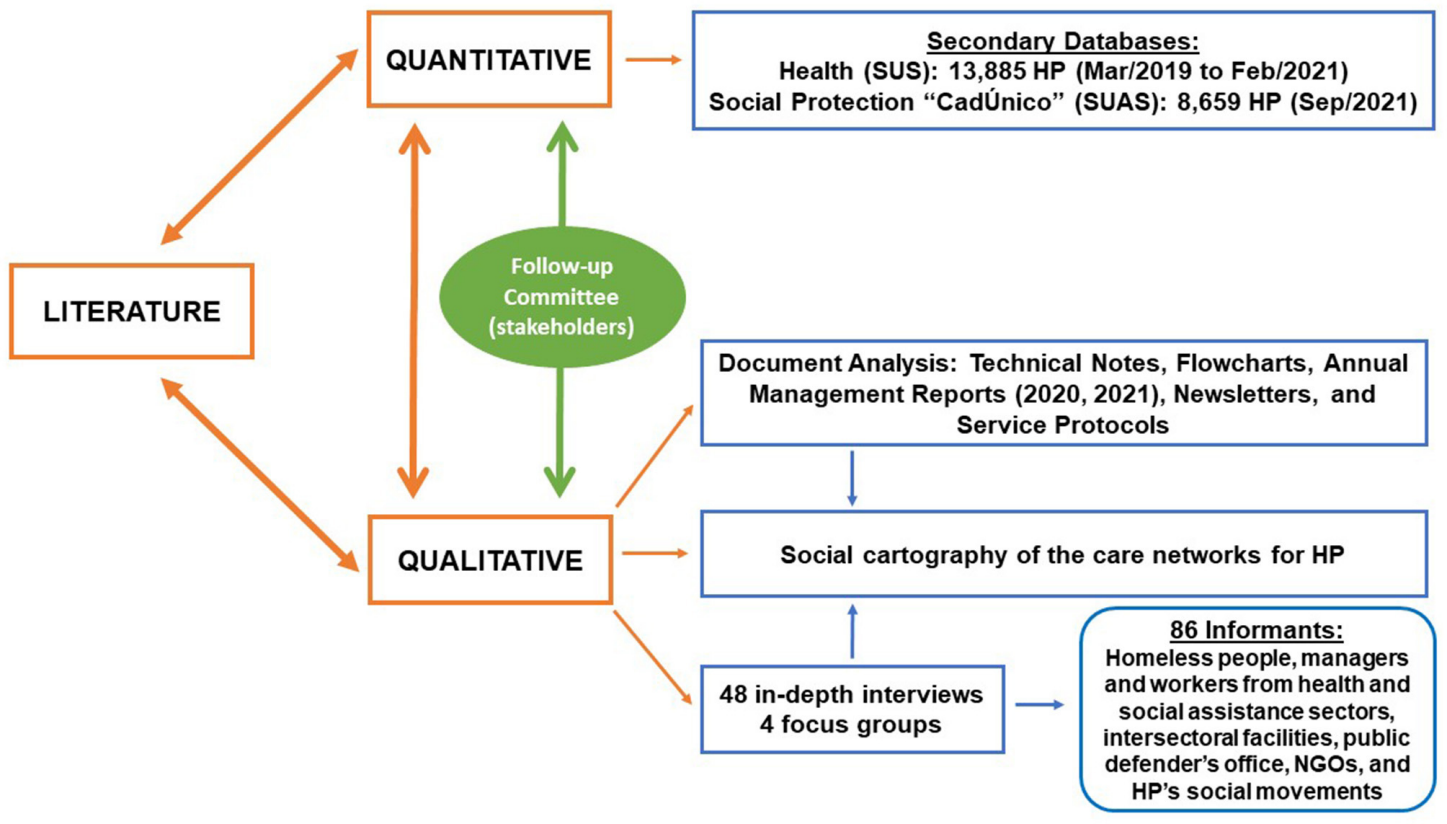
Methodological triangulation. Source: authors.

Quantitative secondary data from government databases on health and social protection, qualitative secondary data from administrative documents, qualitative primary data from interviews and focus groups with representatives of HP, administrators, staff, and others were collected simultaneously and triangulated. Each method will be discussed separately. Figure 1 provides a flowchart with the mixed data collection stages that led to the triangulation of methods.

Added to the triangulation of methods was the creation of a Follow-Up Committee, active during the research, consisting of: (i) health and social protection administrators and staff from various areas and with various roles; (ii) representatives from the National Pastoral of the Street People (known in Portuguese as *Pastoral Nacional do Povo de Rua*), a civil society organization with a historical influence on policies for HP; and (iii) representatives of the National Homeless People's Movement. The inclusion of this heterogeneous committee aimed to correlate the literature with the contextual reality in the field, map the various activities in the territories, mitigate biases, language barriers, and administrative processes, discuss, qualify, and contrast the interpretations of contexts and analyses, and produce an understanding of the results in dialogue with the field of practice in care for HP in the municipality.

The convergent design recognizes the strengths and weaknesses of the quantitative and qualitative methods when assessed separately to expand the theme's complexity and strengthen the results' methodological backing (16), as discussed in detail at the end of the discussion. The breadth and density of the quantitative secondary data from the respective government databases allowed an objective and broad direct visualization of the longitudinal evolution in the target population's profile and the care provided during the pandemic and its trends. Meanwhile, the qualitative sample allowed greater contextualization of the processes, practices of care, and various subjectivities experienced during the same period, while adding qualitative depth to the crosscutting relations between the variables found in the results.

### Data collection

The data that comprised the quantitative and qualitative analyses were collected with different timeframes, according to the study's analytical objectives in its quantitative and qualitative stages.

#### Quantitative

For the quantitative methodology, there were time differences between the health services data and the social protection data. For health, we adopted the timeframe from 1 year prior to 1 year after the start of the pandemic (2019–2021) to allow an evaluation of the municipal health service's resilience in the face of the health emergency. For social protection, we selected the month with the last update to the social protection database called the Federal Government's Single Registry for Social Programs (known in Portuguese as CadÚnico) in the study's target timeframe (September 2021).

The information used in the quantitative stage referred to the following services: (i) for health services: Health Centers, including data from the Street Outreach Clinic teams (a roving primary care service), Mental Health based on information from the Mental Health Referral Centers (known in Portuguese as *Centro de Referência em Saúde Mental*—CERSAM) and Referral Centers for Mental Health Related to Alcohol and Other Drugs (known in Portuguese as *Centro de Referência em Saúde Mental Álcool e Drogas*—CERSAM-AD), and Urgent Services and (ii) for social protection services: the CadÚnico, the unified enrollment system for social benefits.

The scarcity of specific secondary data on the homeless population posed a significant barrier to robust quantitative analyses. One initial obstacle involved the use of tags to categorize homeless individuals in health databases. The first database that was analyzed revealed an unusually high contingent of HP under 5 years of age. Given this scenario, meetings were held with the study's Follow-up Committee, leading to the decision to approach health care professionals on the correct application of the homeless tag. A new dataset was later received for analysis, with the corrections to the previously detected inconsistencies (this dataset was used in the study).

The CadÚnico registry showed a predominance of records with outdated data. The evidence indicated that given the homeless population's mobile nature, part of this dataset was no longer representative of the group. In the process of linking various databases, for example, only 5.6% of the individuals whose records had been updated more than 48 months before were found in the other databases, in contrast with a proportion of 55.0% of the records updated within the previous 18 months. We thus opted to limit the performance of statistical tests to individuals with data updated within the previous 18 months, which coincided with the pandemic, since this database is from September 2021.

#### Qualitative

For the qualitative methodology, we adopted two timeframes for the data collection. The document collection included public documents produced by the municipal government with a focus on the health and social protection departments, where the timeframe covered from March 2020, the month in which the municipality declared the pandemic, to December 2021, the period with the largest accumulation of documents on management of the pandemic in the city. The collected documents included national and local laws and guidelines, technical notes, annual management reports, patient flows, clinical protocols, patient follow-up, and other relevant public documents published on the website of the Belo Horizonte Municipal Government. The qualitative primary data collection, through interviews and focus groups, took place from June 2021 to June 2022 due to the restrictions imposed by the pandemic on in-person activities. Key informants were selected for the interviews and focus groups through the snowball sampling method until the researchers identified data saturation (17). The snowball method was chosen for its capacity to reach hard-to-reach groups (18) such as HP. Primary data were collected with 48 semi-structured interviews and four focus groups, totaling 86 interlocutors during the above-mentioned period. The interviews were conducted with HP, persons with experience living in the streets, staff and administrators from health and special social protection services, a representative from the office of the public defender, and members of organized civil society with initiatives acknowledged by the municipal government in care for the homeless population. The four focus groups were conducted with HP at different locations with the aim of identifying their experiences with access to public services and actions in the response to the pandemic, besides their survival strategies. At the start of the qualitative stage, we conducted preliminary fieldwork to understand the territory's dynamics and the services attended by the HP, which included visiting 17 services, either formally planned as such and/or those viewed as references by the HP. The scripts for the semi-structured interviews and focus groups were developed by the researchers and validated by the study's Follow-up Committee.

### Data analysis

#### Quantitative

In the quantitative stage, epidemiological statistical methods were applied to assess the profile of HP in the municipality, as well as the profile of care supplied to them in health and social protection, using the R software. Given the lack of a unified municipal or national information system on HP, the data were assessed individually and jointly based on probabilistic database linkage. The sizes and time windows assessed in the databases were: (i) health database: 13,885 unique HP from March 2019 to February 2021; (ii) the social protection database, called the “CadÚnico:” 8,659 HP registered in September 2021.

The study variables for the analyses were: (i) health: consultations performed 1 year before and 1 year after the start of the pandemic, evaluated according to the number of consultations and classified according to the International Classification of Diseases (ICD-10) and characterization of the HP treated by the health services according to sex at birth, race/skin color, and age; (ii) social protection: identification of the HP registered in the CadÚnico and their characterization according to sex at birth, age, schooling, duration of homelessness, and per capita family income.

The descriptive analysis used contingency tables, which allowed comparing the distribution of the analytical variables according to groups of individuals. Considering only persons with up-to-date enrollment in the CadÚnico database, i.e., within the previous year and half, that is, up to date during the pandemic, we evaluated the differences in their profile according to duration of their homelessness (1 year or less vs. more than 1 year) using the chi-square test with 5% significance.

The pandemic's impact on the number of health consultations for HP was evaluated through interrupted time series analysis (19). Since the data on the number of patient consultations displayed overdispersion, identified by the Cameron and Trivedi test (20), a quasi-Poisson regression model was adjusted with the covariables time in months, occurrence of the pandemic (yes, no), and interaction between them. The model's fit was assessed by the deviance residuals' normality and the residuals' autocorrelation and partial autocorrelation functions. Based on the results, graphs were produced with the time series of the total monthly consultations predicted by the model considering the pandemic's effect, and the counterfactual, equal to the predicted number of consultations in case they had maintained the same profile as prior to the pandemic.

#### Qualitative

Meanwhile, the analysis of the qualitative data was divided into two lines. The first line was the content analysis through use of the qualitative analytical software ATLAS.ti. The principal theoretical and methodological reference for the content analysis was Bardin (21), who proposes his method as controlled hermeneutic. This analysis has two main functions, one of which is heuristic, enriching the content's exploration and increasing the propensity to discovery, and the other, “proof administration,” which uses the systematic analysis method to verify provisional questions or affirmations. The discursive content is treated by inference or logical deduction of the evident indices, in this case the transcriptions of the interviews and focus groups (21). The transcribed interviews and focus groups were read carefully by eight independent researchers, with no prior definition of categories, oriented by the study's lines: health actions, social protection actions, actions by civil society, and strategies by HP for dealing with the pandemic. The categories that emerged from this reading were consolidated and validated in meetings with the entire research team, and the contents were later reviewed according to the defined concepts.

The second line was the document analysis, based on the principles of contemporary historiography, seeking to explore the documents as representations of realities and narratives. From this perspective, documents are viewed as institutions and thus also as social constructions from a critical and intellectual context that constitute local and temporal demands in their production (22, 23).

## Results

### Profile of homeless people

The quantitative analysis showed that the profile of HP in BH corresponded to the profile identified in the population censuses conducted in the city in 2009 and 2013, as a mostly adult, male, Black population with incomplete primary education (Tables 1, 2). However, although this profile predominated, we identified a trend toward change during the pandemic, with an increase in the number of homeless women and individuals with more schooling. Data from the CadÚnico pointed to a higher proportion of persons with a year or less of homelessness, incomplete secondary schooling (44.8%) or with complete secondary schooling or more (47.2%), white skin color (39.9%), age from 0 to 29 years (54.9%), and female gender (44.8%) (Table 3).

**Table 1:** Profile of homeless people seen at health care units in Belo Horizonte.

**Variables**	**Health care units** ***n*** **(%)**				Total *n* (%)^**^
	**Health centers**		**Mental health**	**Urgent care** ^*^	
	**Outreach clinics**	**Other teams**			
**Consultations**					
Before the pandemic	597 (73.0)	6,865 (68.3)	360 (66.8)	4,110 (72.5)	9,999 (72.0)
During the pandemic	372 (45.5)	5,960 (59.3)	311 (57.7)	2,393 (42.2)	7,707 (55.5)
**For respiratory symptoms**					
Before the pandemic	0 (0.0)	426 (4.2)	1 (0.2)	12 (0.2)	438 (3.2)
During the pandemic	0 (0.0)	612 (6.1)	3 (0.6)	5 (0.1)	619 (4.5)
**COVID-19**					
Tested	0 (0.0)	173 (1.7)	2 (0.4)	0 (0.0)	175 (1.3)
Confirmed cases	0 (0.0)	30 (0.3)	1 (0.2)	0 (0.0)	31 (0.2)
**Sex**					
Female	260 (31.8)	4,136 (41.2)	147 (27.3)	2,264 (40.0)	5,714 (41.2)
Male	558 (68.2)	5,909 (58.8)	392 (72.7)	3,403 (60.0)	8,172 (58.9)
**Race/skin color**					
Brown	532 (65.0)	5,434 (54.1)	340 (63.1)	4,197 (74.1)	8,415 (60.6)
White	104 (12.7)	3,489 (34.7)	89 (16.5)	1,003 (17.7)	4,098 (29.5)
Black	182 (22.2)	975 (9.7)	104 (19.3)	407 (7.2)	1,199 (8.6)
Asian descendant	7 (0.9)	143 (1.4)	8 (1.5)	73 (1.3)	187 (1.3)
Not recorded	0 (0.0)	35 (0.3)	0 (0.0)	0 (0.0)	35 (0.3)
**Age (years)**					
0–15	7 (0.9)	2,535 (25.2)	4 (0.7)	888 (15.7)	3,079 (22.2)
16–40	469 (57.3)	4,093 (40.8)	324 (60.1)	2,774 (49.0)	6,035 (43.5)
41–60	338 (41.3)	2,746 (27.3)	211 (39.1)	1,522 (26.9)	3,770 (27.2)
61 or more	26 (3.2)	854 (8.5)	13 (2.4)	544 (9.6)	1,255 (9.0)
**ICD-10**					
I10—Hypertension	0 (0.0)	504 (5.0)	5 (0.9)	7 (0.1)	515 (3.7)
F19—Psychoactive substance use disorders	28 (3.4)	61 (0.6)	186 (34.5)	0 (0.0)	244 (1.8)
F10—Alcohol use disorders	15 (1.8)	78 (0.8)	173 (32.1)	0 (0.0)	235 (1.7)
F20—Schizophrenia	4 (0.5)	72 (0.7)	93 (17.3)	1 (0.0)	140 (1.0)
Other	809 (98.9)	5,616 (55.9)	420 (77.9)	684 (12.1)	6,589 (47.5)
Not recorded	4 (0.5)	4,394 (43.7)	16 (3.0)	4,978 (87.8)	7,234 (52.1)
**Consultations**					
Before the pandemic	3,844 (51.7)	31,674 (55.5)	4,354 (48.5)	8,099 (65.2)	47,971 (55.9)
During the pandemic	3,593 (48.3)	25,373 (44.5)	4,622 (51.5)	4,324 (34.8)	37,912 (44.1)
Total	7,437 (100.0)	57,047 (100.0)	8,976 (100.0)	12,423 (100.0)	85,883 (100.0)

Source: authors. ^*^Patients treated in emergency may also have been treated in Health Centers and Mental Health and vice versa. ^**^Total of unique patients (does not refer to the sum of the previous columns).

**Table 2:** Descriptive data related to homeless people enrolled in the Brazilian Federal Government's Single Registry for Social Programs (CadÚnico; *N* = 8,659).

**CadÚnico variables**	***n* (%)**
Mean age	42.4 years
Biological sex	
Female	904 (10.4)
Male	7,755 (89.6)
Race/skin color	
Indigenous	0 (0.1)
Asian descendant	38 (0.4)
Brown	5,189 (59.9)
White	1,366 (15.8)
Black	2,044 (23.6)
No information in the database	12 (0.1)
Time in homelessness in relation to last update in the registry	
Up to 6 months	2,518 (29.1)
Six months to 1 year	1,136 (13.1)
One to 2 years	1,014 (11.7)
Two to 5 years	1,664 (19.2)
Five to 10 years	1,112 (12.8)
More than 10 years	1,215 (14.0)
Contact with family members off the streets	
No contact	3,832 (44.2)
Frequent family contact	2,876 (33.2)
Annual contact	448 (5.2)
Monthly contact	1,269 (14.6)
Weekly contact	812 (9.4)
Daily contact	347 (4.0)
Schooling	
None	649 (7.5)
Incomplete primary	4,482 (51.8)
Complete primary	1,246 (14.4)
Incomplete secondary	888 (10.4)
Complete secondary	1,282 (14.8)
Incomplete university	102 (1.2)
No information in the database	10 (0.1)
Per capita family income	
Up to BRL 89.00	8,005 (92.4)
More than BRL 89.00	654 (7.6)

Source: authors.

**Table 3:** Profile of persons enrolled in the CadÚnico registry for less than a year and a half according to the last update (September 2021).

**Variable**	**Time in homelessness in relation to last update in registry—*****n*** **(%)**		**Total—*n* (%)**	*p* ^*^
	**Up to 1 year**	**More than 1 year**		
Age (years)				**< 0.001**
0–29	293 (54.9%)	241 (45.1%)	534 (100.0%)	
30–39	343 (38.5%)	548 (61.5%)	891 (100.0%)	
40–49	279 (29.7%)	660 (70.3%)	939 (100.0%)	
50–59	146 (25.5%)	427 (74.5%)	573 (100.0%)	
60–89	57 (24.4%)	177 (75.6%)	234 (100.0%)	
Total	1,118 (35.3%)	2,053 (64.7%)	3,171 (100.0%)	
Biological sex				**< 0.001**
Female	163 (44.8%)	201 (55.2%)	364 (100.0%)	
Male	955 (34.0%)	1,852 (66.0%)	2,807 (100.0%)	
Total	1,118 (35.3%)	2,053 (64.7%)	3,171 (100.0%)	
Race/skin color				**0.018**
White	203 (39.9%)	306 (60.1%)	509 (100.0%)	
Non-white	911 (34.3%)	1,747 (65.7%)	2,658 (100.0%)	
Total	1,114 (35.2%)	2,053 (64.8%)	3,167 (100.0%)	
Contact with family off the streets				**< 0.001**
Never	457 (34.7%)	859 (65.3%)	1,316 (100.0%)	
Almost never	245 (33.3%)	490 (66.7%)	735 (100.0%)	
Every year or month	199 (32.0%)	423 (68.0%)	622 (100.0%)	
At least every week	217 (43.6%)	281 (56.4%)	498 (100.0%)	
Total	1,118 (35.3%)	2,053 (64.7%)	3,171 (100.0%)	
Schooling				**< 0.001**
None	75 (29.3%)	181 (70.7%)	256 (100.0%)	
Incomplete primary	483 (30.3%)	1,113 (69.7%)	1,596 (100.0%)	
Complete primary	156 (34.4%)	297 (65.6%)	453 (100.0%)	
Incomplete secondary	143 (44.8%)	176 (55.2%)	319 (100.0%)	
Complete secondary or more	256 (47.2%)	286 (52.8%)	542 (100.0%)	
Total	1,113 (35.2%)	2,053 (64.8%)	3,166 (100.0%)	

^*^p-value, chi-square test.

Source: authors. Significant values considering a significance level of 5%.

Meanwhile, the qualitative analysis showed that the pandemic raised new concerns for those working with HP, one of which involved the intersectional vulnerabilities attributed to this population's sociodemographic profile, leaving them more susceptible to COVID-19, besides a consensus among the interviewees that there had been an increase in the number of HP since the start of the health emergency. The main reasons cited in the interviews for this increase were loss of jobs, inability to pay rent due to the country's economic crisis, migration from the interior to the state capitals in search of work, and family conflicts resulting from the increase in shared time indoors.

### Health care

Two days after official confirmation of the first case of COVID-19 in BH, the municipal government published Decree no. 17.304 on March 18, 2020, locking the city down through temporary suspension of activities with the potential for gathering, including commerce and public services. Starting on this date, essential services were defined, and the health and social protection services were reordered according to the new emergency circumstances. All health services were considered essential and continued to function throughout the pandemic.

To contextualize the quantitative results, we begin by highlighting the mapping conducted by social cartography of the network of health care for HP. Primary health care (PHC) services are the preferred portal of entry for users, besides coordinating the care and organizing the flow of care in network format. The principles are universality, comprehensiveness, and equity, and the guidelines are regionalization, territorialization, and person-centered care, among others. Other important portals of entry for HP into the health care system are mental health services, referred to in Brazil as psychosocial care, including the Centers for Psychosocial Care (CAPS, acronym in Portuguese) that comprise the Network of Psychosocial Care (RAPS, acronym in Portuguese) and the Rapid Care Units (UPA, acronym in Portuguese) in urgent and emergency care that comprise the Network of Urgent and Emergency Care (RUE, acronym in Portuguese).

The health care system in BH features a broad primary care network that includes 152 Health Centers and various modalities and health teams with 595 family health teams, 310 oral health teams, and 152 mental health teams distributed across the city's nine regional health divisions. The Network of Urgent and Emergency Care consists of nine Rapid Care Units. As for the Network of Psychosocial Care, the city has nine Mental Health Referral Centers, five Referral Centers for Mental Health Related to Alcohol and Other Drugs, and one Urgent Psychiatric Care service.

Besides the health services at fixed locations, BH has roving PHC services in the Street Outreach Clinics and the program called “BH Joining Hands Against AIDS,” which features mobility to reach users on the streets in sensitive locations, such as public drug use scenes and hard-to-reach areas such as under street overpasses and other unconventional gathering places. The documents on reordering of the services during the pandemic and the interviews point to these services as portals of entry into the health care systems for persons that experienced homelessness recently during the pandemic.

The quantitative analysis of the health database pointed to an overall decrease in care, totaling 85,883 consultations for HP, 47,971 of which before the start of the pandemic (representing 9,999 individuals) and 37,912 after the start of the pandemic (representing 7,707 individuals). The downward total numbers of care reflect the decrease in individuals seen mainly in PHC (Health Centers and Street Outreach Clinics). In the urgent services, before the pandemic, 4,110 HP were seen in 8,099 consultations, while during the pandemic 2,393 HP were seen in 4,324 consultations, that is, a decrease of 41.77% in persons seen and 46.61% in consultations (Table 1).

The most prevalent diagnosis was hypertension, despite a 1.13% decrease during the pandemic. However, the evaluation stratified by type of service revealed different profiles of care according to the services' respective expertise. The highest prevalence rates in the Street Outreach Clinics and Mental Health Referral Centers and Referral Centers for Mental Health Related to Alcohol and Other Drugs were for mental and behavioral disorders involving multiple psychoactive substance use, alcohol-related disorders, and schizophrenia (Table 1).

**Figure 2:**
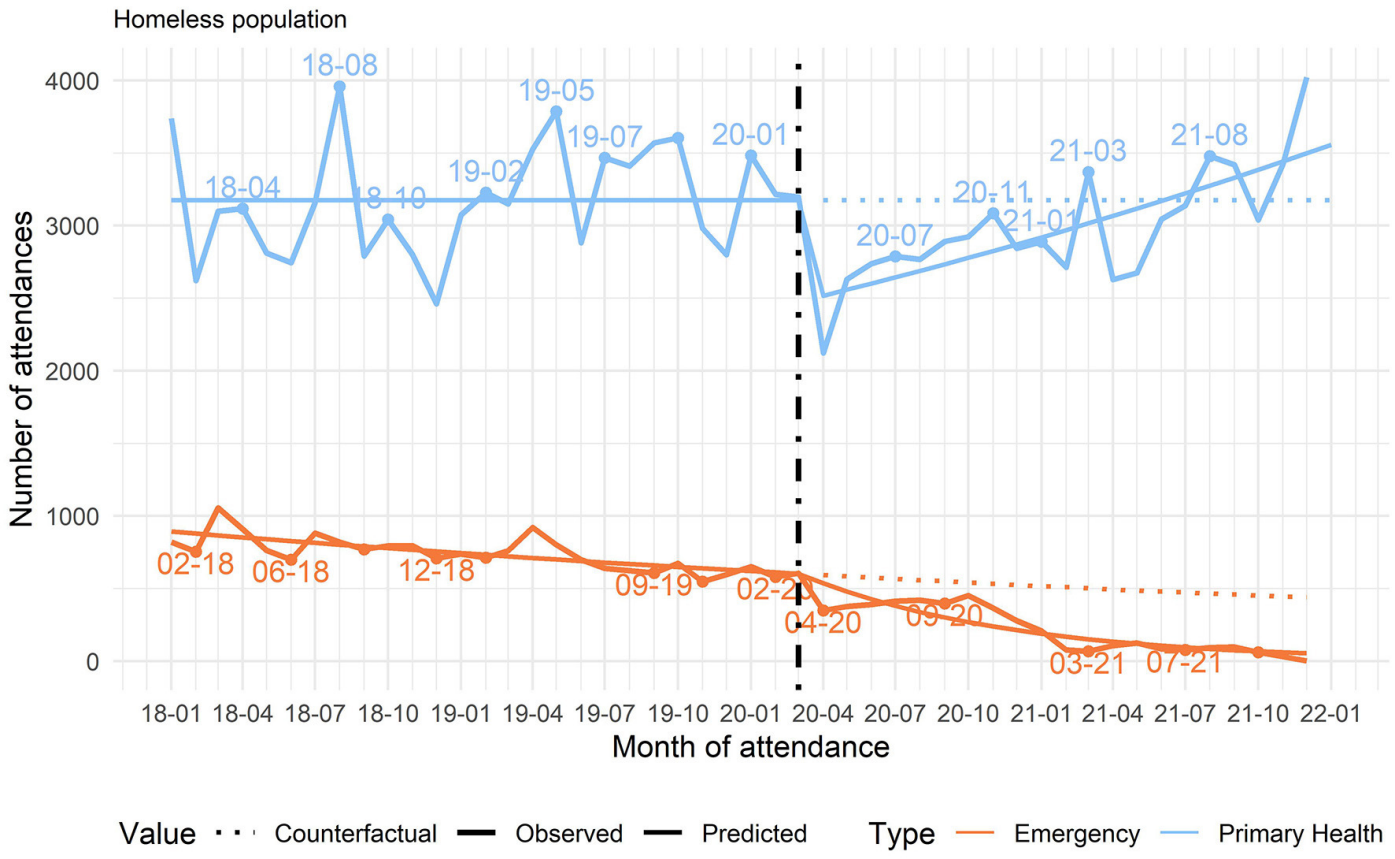
Changes in the monthly number of patient consultations in primary care (Health Centers, Street Outreach Clinics, and Mental Health Referral Centers) and in Urgent Care, Belo Horizonte, Minas Gerais, Brazil, 2018–2022. Source: authors.

**Figure 3:**
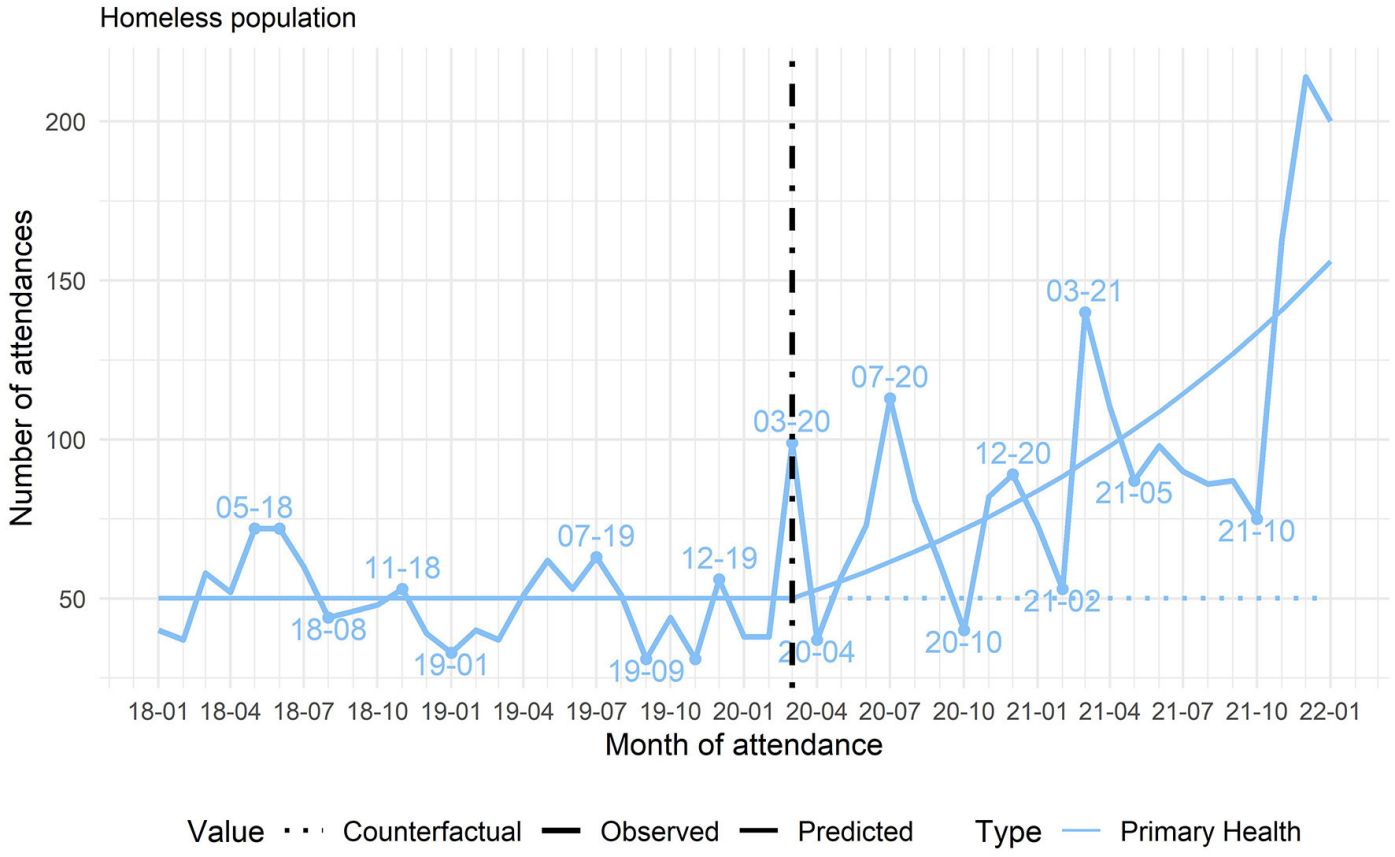
Changes in the mean number of consultations for respiratory symptoms in primary care (Health Centers, Street Outreach Clinics, and Mental Health), Belo Horizonte, Minas Gerais, Brazil, 2018–2022. Source: authors.

Despite difficulties with health care since the start of the pandemic, the health care network succeeded in increasing the number of treatments for HP with respiratory symptoms, with a resumption of the number of treatments for other complaints during the pandemic (Figures 2, 3).

The qualitative data helped contextualize the decrease in the number of consultations. The documents showed that the principal orientation for the general population was to avoid using the health system for mild symptoms or medical conditions other than COVID-19, to avoid causing agglomeration that would increase the risk of SARS-CoV-2 transmission. The interviews indicated that this orientation also tended to keep HP away from the health services, even for treatment of chronic conditions that should not have been interrupted.

The social cartography also indicated that in the early months of the pandemic, the health administration experienced difficulties in reordering the health system, with constant updates to the protocols, but the system's reorganization was gradually consolidated over the months. The health services' eventual readaptation, including stabilization of new flows and guidelines for individuals with respiratory symptoms, correlated directly with the period identified in the quantitative analysis with an increase in care for HP with respiratory symptoms and with the resumption of consultations for other health complaints.

### Reordering of the health care system during peak transmission

Quantitative data showed that health services in general had their activities affected by the peak waves in COVID-19 transmission, testing the limits of the network's capacity. The number of treatments for respiratory symptoms in HP increased by 43.7% after the start of the pandemic (Table 1). The peak treatments for respiratory symptoms occurred in July and December 2020 (Figure 3). We also identified an increase in confirmed COVID-19 cases in December 2020. Importantly, the curve of COVID-19 cases in BH resembles that in other municipalities and countries around the world.

Social cartography emphasized that one of the most important measures in reordering the health care network was influenced by periods with the highest peak transmission of the disease, when the administration determined that each regional health division would have a Health Center temporarily converted into a 24-Hour Non-COVID-19 Health Care Unit, aimed at absorbing the excess volume from the Rapid Care Units. During this period, users enrolled in the urgent care units were referred to other Health Centers. The restructuring deeply affected HP, as it involved the Carlos Chagas Health Center, historically the principal care provider for this group due to its strategic location and established role as a referral hub for HP care, transitioning temporarily to emergency services in Central-South BH. The teams at this unit reported that the temporary change in patient flows hindered care, since it interrupted the ties with frequent users and the effects were still felt after the resumption of regular care.

*We experienced a period at the Rapid Care Units when our patients were divided between three Health Centers, and this interfered with care, understand? This confused our patients, who came here to the Rapid Care Units saying, “I want to speak with Dr. so-and-so, who treated me before.” This created confusion and harmed the patients. The patient would say, “I've been treated here for 20 years!” They had to go elsewhere [for care] which they could do on a day-to-day basis, but when they were forced to go, it got complicated. It was a break in the bond, like, “Oh, right, they're cutting me loose now, when I've been seeing the same doctor for 20 years.”*

Health care worker, Health Center (EGT 011)

### Mental health

The quantitative data also showed an increase in care at the Mental Health Referral Centers for psychosocial care during the pandemic (before, *n* = 4,354, during, *n* = 4,622), with peaks in care in May 2020 (Table 1). Meanwhile, the qualitative data showed that this increase in care related to mental distress was interpreted by health care workers as a consequence of the pandemic, with a resulting economic crisis that in turn led to increased exacerbation of psychosocial crises, as illustrated in the following quote.

*It was very evident that this crisis thing is really a psychosocial crisis, right, an economic crisis. A poverty crisis, not only in the amount of people that began to circulate in the Health Center, which is also real. Our feeling is that there was an increase in the amount of people circulating and experiencing situations of destabilization, it's… psychiatric, you know… psychiatric on the streets. It's not just the amount [of people], I think it's their precarious situation, which became very clear. The patients were increasingly precarious from this social point of view*.

Administrator of a Mental Health Referral Center (EGT 012)

A major difficulty related to mental health, emphasized by patients and health care professionals, was the use of psychiatric medication with its side effects, affecting the patients' treatment. There was a perception of risk among HP that left the users apprehensive toward the use of medicines that might make them drowsy and leave them more vulnerable to violent circumstances on the streets. The supply of medication to reduce abusive drug use and deal with withdrawal syndrome appeared as an important type of care for this population. Health care workers also highlighted the juxtaposition of harmful use of alcohol and other drugs and underlying mental health conditions in some patients.

### Social protection

Social cartography of the social protection network for HP highlighted Brazil's national policy guidelines for social protection, defined as part of social protection, which determines that users in situations of social risk or vulnerability such as HP should be served by the Special Social Protection modality. This is divided into high and medium complexity, but only medium complexity will be included, since high complexity includes sheltering as a condition and has different qualities from the other social care services mentioned.

Medium-complexity social protection adopts as its territorial reference the Specialized Social Work Reference Centers (CREAS, acronym in Portuguese), a regional unit that manages modalities of specialized care for various vulnerable groups, including the Specialized Social Approach Service (SEAS, acronym in Portuguese), a roving service known popularly as Street Approach Outreach. BH has nine Specialized Social Assistance Reference Centers and 102 roving staff in Street Approach Outreach (BRASIL, 2004; PBH, 2022). Another modality features a day shelter service and exclusive care for HP, called the Specialized Reference Center for Homeless People (Centro POP, acronym in Portuguese). In addition to psychosocial care and referrals, the day shelter at the Specialized Reference Center for Homeless People provides facilities for storing personal belongings, restrooms and showers, material for bathing and personal hygiene, laundry, meals, and socialization activities. Before the pandemic, the municipality only had two Specialized Reference Center for Homeless People for adults, with daily capacities to serve 450 and 300 people, respectively.

As for social protection activities, the documents indicated that only some services were considered essential for continuous functioning during the pandemic, but even so their supply of services was restricted by health protocols. The following services continued to function for homeless people: Street Approach Outreach and the Specialized Reference Center for Homeless People. However, the documents and interviews indicated that these services suffered cutbacks in their capacity and vacancies and that the time allowed for people to stay on their premises was reduced due to the COVID-19 protocols.

The cartography of the social protection network identified a major barrier to access by HP, namely the suspension of Specialized Social Assistance Reference Centers in the first 2 months. These are regional referral centers that conduct territorial coordination of a large share of the special social protection network. They later operated exclusively on a remote basis until the 7th month into the city's lockdown. Changes in the supply of social benefits during the pandemic impacted homeless people significantly, especially given this population's new profile. *Bolsa Fam*í*lia*, the principal income transfer program in Brazil, had its entry registry suspended for 4 months by the federal government. Later, the updates to enrollment in the CadÚnico, a prerequisite for the *Bolsa Fam*í*lia* program, were only performed remotely in BH. Despite the implementation of greater bureaucratic flexibility in access to the benefits, the lack of in-person care and the need to use technology for access became a barrier for HP, as reported below:

*We were closed for* ~*7 months [at the Specialized Social Assistance Reference Centers]. Which was a huge, huge mistake in my opinion! And we're still suffering the effects of this mistake. There were people that didn't have access to anything, to information, nothing. They didn't even know that emergency aid existed. Users were ignored if they didn't have a telephone, with no access to technology, with no access to information. There was an absurd increase in homeless people during this period. Why are we still suffering the effects of this? Because there were people that I saw in the spontaneous demand in these last months who had just become homeless, in the first 7 months of the pandemic, a lot of people*.

Social worker, Specialized Social Assistance Reference Centers (EGT028)

The period from November 2020 to January 2021 saw a gradual return to in-person activities in the services. However, due to the new wave of COVID-19 cases in the city, the in-person activities in social protection services established harsher restrictions from January to July 2021. The complete resumption of in-person activities and regular functioning of social protection services only happened in December 2021.

There is a shared recognition among administrators and staff in various services that the social protection services that maintained the in-person format in the Street Approach Outreach and Specialized Reference Center for Homeless People had difficulty meeting the growing needs of HP given the partial lockdown of other social protection services and other services and civil society initiatives. The following quotes express these difficulties:

*You know that situation when you go somewhere and see all those people crowding around? We didn't even know what we were doing, and everybody was begging for help. Because the food donations dried up, everybody disappeared, and they were left out on the streets, with no place to stay, because they had none, so they would go to the Specialized Social Assistance Reference Centers. The Specialized Social Assistance Reference Centers only had us [Street Approach Outreach] (…), the team couldn't go out into the territory, it couldn't, because we were already treating them all there, non-stop. So, I said to the team, what are we going to do? We treated people to the limits of our possibilities, and later, since the demand never stopped inside the Specialized Social Assistance Reference Centers, we had to take the [Street Approach Outreach] team out of the Specialized Social Assistance Reference Centers and allocate them elsewhere to be able to go out on the streets*.

Administrator, Street Approach Outreach (EGT033)

*There were all kinds of difficulties! We had to work without the network. The only network was the health network, and even so it was extremely saturated. And the services soon devised ways to conduct things online! But how are homeless people supposed to do things online? We had to make calls from our cellphones*.

Staff worker, Specialized Reference Center for Homeless People (EGT018)

Meanwhile, given the increase in demand, the social protection administration expanded its medium-complexity services. A third Specialized Reference Center for Homeless People was created in 2020, plus the expansion of another Specialized Reference Center for Homeless People to serve 600 people per day in 2021. The administrators emphasized that although these expansion plans for the services had been considered before, the pandemic accelerated their funding and deployment.

### Facilitators of inter-sector integration

According to the conjunctural analysis from the cartography of the networks of care, despite the initial difficulties in the networks' reorganization, the existence of a greater shared concern over the increased vulnerability of HP during the pandemic allowed executing inter-sector actions with greater collaboration between social protection and health administrators and staff, such as active searches for users with positive COVID-19 tests, the vaccination campaign, or even the inter-sector district meetings that were held online with increased participation.

*So, to talk about the network is always sensitive, right? [laughter]. We managed closer cooperation during the pandemic, right? We managed to establish better dialogue, right? With the pandemic, I mean, things were messed up, really messed up, and we managed to establish closer collaboration and linkage, because my feeling is that [normally] each person wants to focus on their own responsibilities, understand?*

Administrator, Specialized Reference Center for Homeless People (EGT 032)

A key source of care for HP involved the roving health and social protection services, aimed at reaching the target public more effectively. These services managed to maintain the bonds with HP in their territories (often hard to reach), even during the periods with the harshest health restrictions and the city's lockdown. There was a consensus among interviewees that the city's emergency lockdown created barriers for HP to access their minimum necessities, since their survival hinged on actions related to movement in the city, such as donations, work, permission to use private installations and resources, etc. The roving teams' work was thus particularly important because of the distribution of supplies to meet this population's basic health and food needs, especially in the initial months, during the standstill in the city's services to support HP and the reduction in donations from civil society.

*I think the first vulnerability we saw in the field work was food, food security. People starting voicing complaints and demands that hadn't existed before. Like, “I'm hungry, really starving.” So, I think [serving this food need] was positive, among so many other vulnerabilities, and I think we're going to maintain it in the program. Selfcare, the use of personal hygiene supplies, is going to remain in the program regardless of the pandemic. These supplies to the population are going to continue. Due to the complexity of cases, the linkages in the territories improved considerably*.

Administrator, BH Joining Hands Against AIDS (EGT009)

In addition, the mobility of the services and inter-sector coordination in the distribution of the territories' coverage allowed reaching a share of the homeless population that does not normally use public services The roving services thus served as important public channels for communicating guidelines for dealing with the pandemic and distributing supplies for selfcare and to prevent COVID-19. The administrators of both the health and social protections services also acknowledged that the emergency needs during the pandemic allowed speedier approval of budget resources and various processes for subsequent expansion of the services.

### Barriers to inter-sector integration

The results from the cartography of networks of care showed that despite the initial quick reaction by the municipal government, various administrative difficulties emerged during the pandemic. The constant announcement of different guidelines and recommendations with new technical bulletins or updates to the old ones led to disorganization of the work flows due to the difficulty in adapting the services. Staff members reported difficulty in communicating with administrators and insecurity in relation to the protocols, while the administrators reported that this resulted from the speed of the pandemic's spread and the initial lack of knowledge concerning the disease.

In addition, the Municipal Health Department was designated in the 1st week as responsible for helping with the protocols and health training for workers in the Social Protection Department, while there was a 3-month delay in extending the same training to the workers dealing with HP. This delay led the social protection services to make their own health decisions to deal with pandemic, besides jeopardizing health-related communication with homeless people themselves, due to discrepancies in the recommendations. Even so, there was a shared perception between the health and social protection sectors concerning close collaboration as the pandemic unfolded, resulting in more constant recommendations and intensity in the inter-sector actions, including shared management of the Emergency Sheltering service.

On the other hand, a major barrier to integration of the services supply was the lack of a shared information system among public services, especially between health and social protection, which provide care according to the objectives of the National Policy for Homeless People (according to which they are supposed to be integrated). There was also major difficulty in sharing data between various social protection services, which in turn affected the network's integration since the civil society organizations mainly administered the services using their own information systems.

The difficulties and barriers for health care included such aspects as ignorance of inter-sector flows, red tape, users' lack of understanding of protocols, lack of transportation to health services, high turnover of personnel, occasional absences of physicians, perception of health care workers' prejudice or disrespect toward HP, and users' impatience with waiting time for care. Issues involving lack of ID papers also appeared as a barrier to accessing health services, although there is a specific ruling exempting homeless people from presenting any kind of documentation to be treated. The pandemic also created difficulty in accessing medicines. Health professionals reported that the repurposing of Health Centers as Rapid Care Units caused problems with the distribution of medications, since users were unable to receive medicines in some centers that they had used normally as their references and had to turn to other centers.

### Inter-sector innovation: COVID-19 emergency sheltering

The social cartography on the networks of care showed that the health emergency created space not only for reordering public services, but also for innovation. The public concern over COVID-19 transmission among homeless people included public debates (and other discussions inside the services' administration) on their treatment and their inability to social distance on the streets. A new inter-sector service was thus launched on April 7, 2020, administered jointly by the Health and Social Protection Departments, called “Temporary and Emergency Sheltering Service for Homeless and Other Socially Vulnerable People.” Flows were established for screening and referral of people with respiratory symptoms and suspicion of COVID-19, but without clinical indication for hospitalization. Access to Emergency Sheltering was exclusively via referrals by the health services, after evaluation of symptoms, without allowing direct access by users and/or referrals by social protection services. The services that referred users were Rapid Care Units, Health Centers, Street Outreach Clinics, and the municipal program Belo Horizonte Joining Hands Against AIDS. In 2021, about 70% of the referrals came from the Central-South Rapid Care Units, which implemented the Specialized Center for Patients with Suspected Coronavirus Infection (CECOVID, acronym in Portuguese), concentrating care for individuals with respiratory symptoms, besides other specialized COVID-19 services at the beginning of the pandemic.

Emergency Sheltering initially offered 260 vacancies in a hotel building (SESC) for 14 days of social distancing. The service operated at this location until August 28, 2020, and was transferred that September to a smaller hotel with just 22 places. In both cases the service was located outside the city's central areas (which have the largest concentration of homeless people), so they had to travel long distances to reach it (they saw this as a barrier). Meanwhile, the staff reported overuse of the Specialized Center for Patients with Suspected Coronavirus Infection and emergency sheltering by users who wanted to spend a night in the shelter with hotel-style facilities. This overuse occurred particularly at the beginning of the pandemic, when the criteria for referral were exclusively clinical, since rapid tests were still not available and the turnaround time for COVID-19 Reverse transcription polymerase chain reaction (RT-PCR) results was more than a week, given the heavy demand in the municipality.

***R1:*** *(…) on the one hand it was bad, since the place [a hotel unit belonging to SESC] was cool, so people started showing up too often …*

***R2:*** *Misusing the service*.

***R1:*** *Pretending to be ill*.

***R2:*** *Especially on weekends, couples…*

***I:*** *Didn't they do the [COVID] test here?*

***R1:*** *They did, but it took a long time to get the result back*.

       R1 and R2: Social workers, Specialized Center for Patients with Suspected Coronavirus Infection (EGT004)

       I: Interviewer

Even so, administrators reported that the decision to change the location and reduce the number of vacancies was based on the lack of demand, since there were fewer COVID-19 cases in homeless people than initially predicted and the pandemic's follow-up indicators had improved. In fact, the health care staff reported that there were few cases in proportion to the general population. The purported reasons were isolation of HP from close contacts with the general population due to stigma and the fact that they circulated in the open air. The emergency service was transferred in July 2021, this time to a health unit, UAPI Barreiro, with a supply of 30 vacancies. This move from hotel installations to a hospital unit was identified by the unit's staff as an important reason for the decrease in the spontaneous demand by symptomatic homeless people at health services and the greater evasion from the unit during lockdown. In addition, during the period of the move from the first location, a more efficient testing flow had already been organized, allowing a more rapid response for individuals with symptoms and thus shorter waiting time for cases with negative results.

***R2:*** *Barreiro is more like a hospital, an isolation unit today. Not like the SESC [hotel]. At the SESC, there was a cable TV, refrigerator bar, and cold drinking water, so it was like a party. Some people even went there as many as 12 times*.

***R1:*** *They would be discharged and return immediately to the Rapid Care Units. And they would go to the Rapid Care Units to ask to come back [to the hotel]*.

***R2:*** *We had a lot of cases like that, but at Barreiro, now, we only get people who really are sick*.

***R1:*** *And when we linked with the health [department], the results started coming back fast. [The patient would say]:“But I've only been staying here for 2 days!” They thought it was odd, because at the SESC it took longer for them to be discharged*.

R1 and R2: Administrator and worker, Emergency Sheltering (EGT013)

The evasion of homeless people during lockdown was a source of conflict between health and social protection during administration of the emergency service. Users had difficulty adhering to the 14 days in isolation, considering the service's rules, such as abstinence from alcohol and other drugs and the ban on pets. Evasion was a major issue in both individual care (because of interruption of treatment) and collectively, because of COVID-19 transmission (users were prohibited from accessing other public services until returning from treatment). The following quote from a mental health administrator explains that part of the conflict between the health and social protection sectors resulted from differing views on the practice of necessary care for HP pertaining to their mental health and substance abuse vulnerabilities.

*The person would come and ask [to stay] as a mental health [patient] or even as a homeless person, and if they got a little rowdier, mental health wouldn't let them stay. And they had a flu syndrome, and was I supposed to leave them in the Mental Health Referral Center? The person was in a [psychiatric] crisis, understand? Where was I going to send this person? It they were unable to stay, this stimulated evasion. Especially these people*.

Administrator, Mental health (EGT003)

Evasion was particularly high in homeless people with mental distress. There were also barriers to the use of medication due to difficulties in managing mental health crises during isolation. A flow was later created for health units to dispense prescriptions and medications to patients in Emergency Sheltering.

### Inter-sector innovation: inter-sector care at the day shelter

Finally, there was important agreement among users, staff, and administrators in the health and social protection sectors on the significantly positive impact of initiatives by the civil society organization Pastoral of the Street People, which conducted systematic activities in care and referrals, promoting integration between the health and social protection networks, mobilizing other organizations and government agencies to care for HP during the pandemic. Two main activities stood out: the installation of a day shelter and inter-sector services in the city center called the “Emergency Street Corner” and housing facilities for temporary shelter and psychosocial support for HP.

Participants in the Emergency Street Corner had access to nursing and social protection services, which provided orientation and referrals to the various networks, besides in-person participation by the roving health services and Offices of the Public Defender and Public Prosecutor. The initiative also provided a series of inter-sector activities such as registration for accessing services and benefits, distribution of food and donations, shelter and care for pets, workshops and work orientation, classes, artistic activities, and socialization, besides showers, toilets, running water, and a laundry. Health and social protection users, staff, and administrators all identified this service as an important reference and source of support in care for health and social protection for HP during the pandemic, as seen in the following quotes:

*Thank God this emergency street corner came, but now they're trying to take it away from us. It's a big necessity, you know, because the street corner meets a lot of our needs. They help us with everything. You can come any day of the week and there's a social worker there with everything, see? There's nothing like it elsewhere. The city government doesn't work right. They reduced the staff, they reduced the hours, they cut back on a lot of things. And now there's talk about closing this down, too, and we're all asking each other, what are we going to do?*

Homeless person (EPSR03)

*You can see how much support we're getting, right? People are taking showers, people are doing their laundry, people are eating, people are making handicrafts at the workshop. All this keeps people busy, keeps their minds occupied. I think that more of the Street Corner and activities like it are needed, regardless of whether it's the Pastoral of the Street People or the government. They shouldn't even think of shutting down a place like this*.

Homeless person (EPSR04)

*The Street Corner was set up there, which, my God, was a huge relief, because homeless people had somewhere to go, a space to stay, because they didn't have a place. This helped us a lot, because [HP] concentrated there, and [the staff at Emergency Street Corner] contacted me regularly. Whatever they needed, we would work out an exchange. So, that space benefited all of us. We were unable to cover all the locations, so the Street Corner was a space that truly worked. And the Street Corner is run by the Pastoral of the Street People, which does work in the territory, like we do, too, we as a public policy agency and they as an institution*.

Administrator, Street Approach Outreach (EGT033)

The Emergency Street Corner operated from September 2020 to August 2021 and was the initiative most frequently cited by the various interviewees due to its innovation in integrating services and activities, the need for it to continue, or as an inspiration for the supply of analogous inter-sector services.

Table 4 summarizes a compilation of the study's main results.

**Table 4:** Main results of the study.

**Profile of HP in the pandemic**
Population mostly male, adult, Black, with incomplete primary schooling. However, although this profile was still predominant, a trend toward change was seen during the pandemic, with an increase in homeless women and people with more schooling.
**Health**
There was an overall decrease in patient consultations for homeless people during the pandemic, except for an increase in mental health services. Interviewees attributed the overall decrease to public orientation to avoid crowding in services. Hypertension was the most frequent diagnosis in homeless people. Reordering of the health system was influenced by periods of peak COVID-19 transmission. During this period, users linked to support units for urgent care were referred to other Health Centers, which particularly affected HP, since this broke the bond with frequent users (and the effects were observed even after the resumption of care at the unit).
**Social protection**
A major barrier for access to social protection services by HP was the suspension of Specialized Social Assistance Reference Centers, referral centers that organize regional services (later they only operated online). Changes in the supply of social benefits during the pandemic significantly impacted HP, especially considering this population's new profile. The principal income transfer program (*Bolsa Família*) had its entry registry suspended for 4 months by the federal government. The updates to the CadÚnico registry, a prerequisite for the *Bolsa Família* program, were done exclusively by remote access from this time on in Belo Horizonte. The increase in demand for medium-complexity services due to the health emergency accelerated the plans for permanent expansion of the supply of day shelter services (Specialized Reference Center for Homeless People).
**Facilitators and barriers for inter-sector integration**
Increased shared concern between health and social protection over the increased vulnerability of HP during the pandemic triggered an increase in the volume and intensity of inter-sector actions and greater dialogue between the sectors. The roving health and social protection services stood out for their territorial coordination, guaranteeing maintenance of access and bonds with services for HP, considering their locations and mobility and distribution of supplies for standard necessities and COVID-19 prevention. A major barrier for integration of supply was the lack of a shared information system between public services, especially between health and social protection. Other barriers to care for HP remain as before the pandemic, including the following: lack of knowledge of many of the inter-sector flows; excess red tape in the services, such as denial of access to services for undocumented homeless people; lack of transportation to health services; high staff turnover and occasional lack of physicians; and perceived staff prejudice or disrespect toward HP, among others.
**Emergency inter-sector innovations**
Emergency care for suspected COVID-19 cases showed positive results. However, no organization of a service was identified that aimed to manage mental health crises during the lockdown. Major evasion from lockdown was identified due to harm reduction care for people with alcohol and other drug use. The inter-sector day shelter services at the Emergency Street Corner were extremely important in care for HP: supply of nursing and social protection services; orientation and referrals to the service networks; registration of HP for access to services and benefits; distribution of food and donations; workshops and work orientation; art activities and socialization; showers, toilets, running water, and laundry tanks, among others.

Source: authors.

## Discussion

Contrary to international evidence pointing to neglect for homeless people in situations of disaster and emergency preparedness and response (24), key factors were found in BH that facilitated more equitable access to care during the COVID-19 pandemic. The study's results provided greater evidence for increased access to services by HP in the face of existing barriers, considering real-time adaptations in the reorganization of health and social protection services, increased inter-sector care, expansion of existing services, and the creation of new inter-sector services targeted to HP, such as Emergency Sheltering and the day shelter at the Emergency Street Corner. However, a reading of the literature suggests that many interdependent variables directly affect the situation of HP and their access to health and social protection services.

### Homelessness and its underlying causes

Epidemics and pandemics are predicted to affect the health of marginalized groups more intensely due to the social determinants of health, which include structural determinants such as poverty and discrimination, which in turn influence intermediate determinants such as health, housing, and employment (25). No global study exists on the pandemic's effects on HP, but the 2022 Human Development Index (HDI) (a comprehensive metric that includes health, education, and living standard) reports a significant setback for countries and an increase in inequalities, reverting to the same level as in 2016 (26).

According to the health and social protection databases, the profile of homeless people in BH mirrors the same historical trend found in Brazil's national and municipal censuses for this population group (27–29), and in government registries (30). However, the qualitative data point to agreement among the various stakeholders in terms of a perceived increase in the number of HP since the beginning of the pandemic. According to an estimate by Brazil's Institute for Applied Economic Research (IPEA), there were 281,472 homeless people in Brazil in 2022, or an increase of 211% from 2012 to 2022, with the largest concentration of homeless people in Southeast Brazil, including BH (31). That same year, nationwide data from the CadÚnico database pointed to 236,400 individuals registered as homeless, only 4% of immigrants, i.e., ~1 out of 1,000 Brazilians were homeless that year (30). Economic crises, lack of affordable housing, poverty, unemployment, and family breakdown are considered determinant in the increase in HP in large cities, according to the Brazilian and international literature (32, 33).

The increase in Brazilians living in the streets during COVID-19, including women and people with more schooling, also reflects the country's rising unemployment, political instability, and government disorganization during the pandemic, which affected economic classes that were previously more protected (34). Despite local specificities, homelessness is known to be a global phenomenon that affects an estimated 2% of the world population (32). Not coincidentally, the internationally shared reasons are linked intrinsically to capitalist modernity which, through the drivers of economic progress and globalization, creates “growing masses of ‘wasted humans”' that it is incapable of reassimilating or annihilating (35).

In Brazil, there is a relationship between the phenomenon of homelessness and the country's socio-historical formation. In this context, it is important to consider the extensive period of slavery and its transition at the end of the nineteenth century. Under the myth of the free African, the freed black population were “thrown onto the streets to fend for themselves like undesirable human waste” (36) in precarious living conditions without concrete possibilities for social and economic inclusion. Added to this, they were targets of a criminalization process that affected those who were jobless and considered idle, with black and poor people being the main targets (37). On these foundations, an unequal society was established (38) sustained by structural racism (39). The combination of these elements is reflected in the fact that the HP is composed of ~78% (30) black individuals, while in the general population this number is about 55% (2). Furthermore, it is observed that health indicators in the general population are worse among the black individuals when compared to white individuals, as is the case with morbidity and mortality rates (40). Additionally, among people in homelessness, there is a considerable statistical difference between white and black individuals in terms of illiteracy, education, and time spent living on the streets, with black individuals presenting the worst rates (41).

### Challenges in accessing care during the pandemic

Many studies have identified adequate permanent housing (absent for HP) as an important social determinant of health (42), while illness and lack of care related to unhealthy behaviors have also been identified as factors for loss of housing (43). The homelessness phenomenon is thus often associated with worse health, higher rates of acute and chronic diseases, and higher mortality rates (44).

In BH, the break in continuity of care, as reflected by the decrease in the number of treatments for HP immediately after the start of the pandemic, was a factor in this group's acute-on-chronic vulnerability, with difficulty reestablishing ties to return to care, also observed in Brazil's general population (45, 46). Although there is an overall national trend, the status of homeless people is known to deserve special attention, as a population naturally susceptible to symptomatic infections due to their increased risk of environmental exposure, greater risk of hospitalization, exacerbation, and death, accelerated physical decline, and mental health problems sometimes associated with alcohol and drug use. Homeless people also suffer great social vulnerability and face huge barriers to access the health and social protection systems, a situation exacerbated by the pandemic. Thus, with or without COVID-19 or another future pandemic, the decrease in care by the health system for these individuals is worrisome, with major implications for their health conditions (47–50).

An emergency intervention that directly harmed HP was the city's general lockdown (even with the counter-response through the distribution of supplies by the roving services and maintenance of health services) since it raised barriers to meeting basic needs. Consequently, the lockdown may have affected the prioritization of HP even more and negatively impacted selfcare in health. This is based on evidence in the literature that homeless people may have different healthcare-seeking behaviors than the general population, emphasizing: (i) their prioritization of basic needs such as food, clothing, and shelter rather than health; (ii) postponing the search for services until serious aggravation of their health condition; and (iii) mistrust of health services due to stigmatization, physiological difficulties, and emotional stressors (6).

The literature also corroborates the findings that barriers (both systemic and those related to the pandemic) to accessing health and social protection services can lead HP to feel stigmatized. These barriers add to those considered “contextual,” such as staff attitudes and perception of access, documentation, lack of knowledge, transportation to health services and long waiting time, causing evasion or dropout from care and follow-up (6, 51–53). This was further aggravated by the structural barrier involved in homeless people's difficult access to digital services (due to the pandemic's circumstances, access to many social benefits was only regularized online). This exacerbation of homeless people's exclusion was also seen in other cases with the online transition of health and social services previously provided in person (25, 26, 54).

### Inter-sector responses, resilience, and innovations

Even so, the health system showed its resilience and capacity for inter-sector rearticulation with the special social protection system during the pandemic, even after the initial decrease in care. There was an increase in care for homeless people with respiratory symptoms, a priority at moments of peak COVID-19 incidence, with a resumption in the number of treatments for other factors even during the prevailing pandemic (Figures 2, 3). The continuity of care in mental health, the inter-sector links that emerged during the pandemic, and the reorganization of services to comply with the health safety rules also showed their capacity for resilience. Another study in BH corroborated the importance of inter-sector linkage between health and social protection for increasing the flows of respiratory patients and access by HP to social distancing (3). The health systems' resilience was an important factor for continuity of care and the guarantee of rights (55).

The major increase in mental health care during the pandemic (including alcohol and drug treatment centers) differed from the other results and signaled the capacity of these services to absorb the increased demand. One can infer that the increase in care may have resulted from the increase in the number of homeless people during the pandemic and the fact that this population was more exposed to exacerbated vulnerability and decreasing care in other services, with a direct impact on these people's psychological distress. According to an integrative review on access by HP to the Network of Psychological care in Brazil (56), studies have identified limitations in public policies for mental health targeted to this group. The increase in mental health care is even more atypical if one considers the evidence that health services restrict the access, fail to provide adequate support, and fail to meet the specific needs of homeless individuals with mental disorders, a situation observed in other studies in BH (57). Other studies confirm the hypothesis raised by health care staff that the increase in the amount and severity of mental distress during the pandemic is related to the pandemic's context (58–60). In addition, homeless people were identified as one of the population groups most vulnerable to exacerbation of mental health conditions during the pandemic (61), possibly explaining the increase in their care.

The study also showed increased capillarity of the networks of care through greater constancy and intensity of inter-sector action between health and social protection during the pandemic, which included more frequent inter-sector communication, referral, and user follow-up. Strengthening inter-sector care has emerged as a possibility for solving complex problems affecting populations. Based on an integrated view of users and social problems, systems should attempt to overcome policy fragmentation in response to demands that extrapolate single social policies (62). However, in the current case, there was no proper regulation or systematization of this inter-sector collaboration, built on an emergency basis during the pandemic.

The importance of inter-sector care adds to the challenges of treating various vulnerabilities in this specific group. The increase in homeless people can be understood as a complex social phenomenon that requires a high degree of linkage between policies, sectors, and social actors, where isolated specific programs are insufficient, considering the interdependent social, housing, health, safety, and other challenges (25, 43). In Brazil, the literature on social policy management based on inter-sector care emphasizes the need for operationalization of the concepts of equity, decentralization, territory, networks, and social rights (62).

The Emergency Street Corner initiative, as a temporary inter-sector service, showed the capacity to deal simultaneously with interdependent challenges for homeless people. It also became a positive example based on its capacity for rapid linkage with the health and social protection networks, even though it was not a service planned in typical public services. Its concentration of inter-sector services and users in the same location intensified the communication and flows with other services, thereby improving the inter-sector follow-up of users and increasing the intensity of inter-sector referrals during the pandemic.

The territorial reach and equity of access to public services underscore the essential work of roving health and social protection services and their inter-sector territorial mapping to guarantee greater access by the population to the itinerant services, even in locations with more restricted access. In addition, the services were important public channels for health promotion and orientation to deal with the pandemic. Health promotion advocacy is a successful alternative for psychosocial issues, with the potential to alter the pattern in the search for help by HP and reduce the burden of work for primary care teams (63).

The creation of new inter-sector services during the pandemic, such as Emergency Sheltering and the Emergency Street Corner, further indicated the capacity for innovation in the face of the health emergency, but consistent with preexisting needs in vulnerable populations (64). The innovation in public efforts driven by the pandemic and the provision of new facilities for HP was also observed elsewhere in the world in various cities with different configurations and coverages, while noting a trend toward sudden and unusual funding for this public (12, 65, 66). These initiatives should not be lost. An opportunity thus emerges to take advantage of the recently created structures to expand the coverage for other transmissible and epidemic diseases, because the lack of housing for homeless people makes them proportionally more vulnerable to a series of diseases, as exemplified by recent outbreaks of typhus, hepatitis A, tuberculosis, trench fever, and *Shigella* among HP in the United States (11).

The current study proved the hypothesis in the literature that chronic physical and mental health conditions and substance abuse by some homeless people are obstacles to social distancing and treatment for COVID-19 (11). The difficulties with evasion and dropout from Emergency Sheltering provided an opportunity to rethink the prevailing model, especially considering the initial difficulty in dealing with individuals with abusive or chronic drug use, who normally have greater difficulty accessing services due to negative and stigmatizing experiences with staff (51). Alcohol and drug abuse is also a permanent issue that requires expanding the supply of services for HP, since the literature identifies such abuse as both a potential contributing factor and consequence of homelessness (32).

On the other hand, the expectation that HP would be a specific risk group for COVID-19 (11, 67) was not proven in BH. There were fewer cases among homeless people than in the rest of the population, attributed to isolation from close contacts with most of the population due to stigma and the fact that they spent most of the time in open spaces. Studies corroborated the hypothesis that these conditions favor less contagion, noting that SARS-CoV-2 is transmitted predominantly by the airborne route in close quarters (68, 69), with shared rooms as the main cause of superspreading (69) and much lower risk of infection in outdoor environments (70).

Even so, the investment in (and acceleration of) municipal plans for expansion of social protection and health services showed that the pandemic acted as a factor reorienting the priority and deployment of more funding for such services. In 2020, the Brazilian federal government allocated some BRL 635.5 billion (US$ 121 billion) in budget funds to fight the COVID-19 pandemic, with BRL 113.5 billion (US$ 20.6 billion) transferred to states and municipalities (71). In addition, preliminary data from the WHO (72) on public expenditures in health show that the pandemic induced a new world record. In 2020, global health expenditures reached US$ 9 trillion, the equivalent of 10.8% of global GDP. Yet the investments were highly unequal in international terms, with high-income countries accounting for some 80% of total spending. A large increase was also seen in per capita health and social protection expenditures in upper middle-income countries. However, the limitation of available data still prevents a conjunctural analysis of expenditures in most countries during the 3 years of the pandemic (72).

### Study limitations and strengths

The study presented some limitations. We were unable to access all the databases in the same timeframes, since the CadÚnico database adopts different logistics for updating, which prevents a longitudinal reading of the data (as done with the health database). Thus, the data may have been outdated in some datasets, both recording the presence of homeless people that were no longer living on the streets and failing to track others whose homelessness was recent. In addition, Brazil's social protection services do not require enrollment in the CadÚnico for care, but only for receiving social benefits, so it was not possible to quantify access to the services. All the information for characterizing HP in services was based on self-declaration of homelessness. This means that the study based on this database had high sensitivity and low specificity. The last limitation involves data on urgent care provided by the BH Municipal Health Department. We did not have access to the data from the main Rapid Care Unit in BH, the Central-South Rapid Care Unit, which prevents us from generalizing the results for urgent care for HP.

The study's strengths featured the database linkage method, which allowed minimizing the data collection challenges for identifying HP (who may be undocumented or choose not to show their ID papers during care at health services or social organizations). In Brazil, the lack of completion of the taxpayer identification number (CPF in Portuguese) or its non-existence in some databases (although it is currently used by the federal government as the standard personal identification document) means that the probabilistic method is extremely important for linking databases produced by different institutions (73). Other strengths were: the unprecedented scope, for Brazil, of methods and data from the same city; the partnership with the municipality of BH, with the availability of large identified databases, which allowed evaluation of data from specific services for HP; the possibility of conducting a longitudinal study based on health data; the study's Follow-up Committee, which allowed identifying biases, qualified the data analysis, and contributed to a productive dialogue on practices of care for HP in the municipality and thus led to subsequent spinoffs in the field. The interviews and focus groups sought to encompass the variations in subjectivities with actors from different locations, roles, and social positions, both administrators, staff, and homeless people as well as representatives of social movements. Finally, the triangulation of methods contributed to a convergent dialogue among different types of data for a more comprehensive understanding of homeless people and the effects of adaptations of policies targeted to this population, aimed at expanding the data on this vulnerable group and learning from the specific case to encourage the debate on practices and policies for them.

## Conclusion

Even considering the difficulties in obtaining precise quantitative data on homeless people and the care for them to provide an exact metric on their access during the pandemic, the quantitative and qualitative evidence collected in this study points to more emergency interventions that involved actions or services that facilitated access, more than barriers. However, when considering the application of these lessons to other contexts, whether in emergency situations or in future daily practice, it is important to recall one of the foundations that sustained their relative success, namely the fact that BH already had robust health and social protection services designed specifically for serving HP. These services sustained the new emergency measures such as an increase in inter-sector meetings and greater ongoing follow-up between the existing services, besides using this structure to increase access, as in the expansion of vacancies in the Specialized Reference Center for Homeless People and Specialized Center for Patients with Suspected Coronavirus Infection for testing, diagnosis, and treatment of COVID-19 in the Rapid Care Units.

The existence of national guidelines for inter-sector care for HP cannot be ruled out as a positive influence, although the municipalities are responsible for implementing these guidelines. It was noteworthy that a health emergency was needed to intensify the relationship between health and social protection services. However, there is a critical need for further development and enhancement in inter-sector protection strategies for this population. This progress should be informed by the coordination challenges encountered at the onset of the pandemic, leading to the creation of robust preparedness and response plans for future emergencies. Such plans must prioritize the systematization of inter-sector collaboration, moving beyond *ad hoc* responses to establish a consistent and integrated approach across different sectors. This systematic approach is essential for ensuring that services are not only effectively coordinated during crises but also become a routine part of the care provided to the homeless population. Sustaining and building upon the inter-sector services developed during the pandemic is crucial. These services have demonstrated their value in addressing the complex needs of the homeless population and should be maintained as part of the standard care framework. The continuity of these services ensures that the gains made during emergency responses are not lost but rather integrated into the normal functioning of social and health care systems. In addition, significant advancement is required in the development of shared information systems. Such systems are vital for facilitating efficient data exchange between health and social protection sectors. By improving communication and information sharing, these systems can lead to more informed decision-making, better resource allocation, and more targeted and effective interventions for the homeless population.

The services with the most positive evidence and least need for infrastructure to be replicated locally feature the roving services in both sectors and their inter-sector care. They foster greater reach due to their mobile territorial coverage, which became the portal of entry for access to networks of care, given the mobility of homeless people and the locations with limited presence of other services, in addition to the distribution of supplies, which help attenuate the exacerbation of vulnerabilities in emergency situations. In addition, the temporary provision of various inter-sector services simultaneously with the day shelter at the Emergency Street Corner was considered a key factor in expanding and intensifying the networks of care for HP in BH during the emergency phase (with plans for the model to be continued and further expanded in the future).

As for the Emergency Sheltering initiative, it is necessary to continue this service's model, including other possibilities of care related to chronic and communicable diseases. It is also necessary to expand the debate on practices of care for homeless people to include a better understanding of their vulnerabilities, especially related to mental health and/or abstinence from alcohol and drugs, which were complicating factors for COVID-19 quarantine and treatment. We thus echo the proponents of equity-oriented health care (EOHC), which aims to acknowledge patients' vulnerabilities and orient their care according to their circumstances and difficulties.

It is also necessary to learn from the challenges and barriers that emerged from the emergency measures. It is necessary to create interventions that mitigate the negative effects of the city's lockdown, during which homeless people lacked access to standard necessities, besides difficulties in access to digital online services. The lack of data and more in-depth studies on homeless people also hinders targeting actions and care for this group. Considering the study's evidence and the literature, according to which homelessness can be influenced by economic crises, unemployment, lack of adequate home loan conditions, family breakdown, and others, further investigation of these possible correlations is important. The understanding of inter-sector variables affecting HP would contribute to better targeting of investments in interventions that work at the root causes of these issues or that increase the effectiveness of health and social protection services to mitigate or deal with such circumstances.

## Data availability statement

The datasets presented in this article are not readily available because, access to the databases complied with the guidelines of the Brazilian General Act on Protection of Personal Data, Law no. 13.709 of August 14, 2018. The researchers are not authorized to share the databases, since these are public health data. Any such authorization should be requested to Belo Horizonte's Municipal Government. Requests to access the datasets should be directed to Health Secretariat of Belo Horizonte.

## Ethics statement

The studies involving humans were approved by Institutional Review Board of the René Rachou Institute, Oswaldo Cruz Foundation (FIOCRUZ), and Institutional Review Board of the Health Secretariat of Belo Horizonte-MG. The studies were conducted in accordance with the local legislation and institutional requirements. The participants provided their written informed consent to participate in this study. Written informed consent was obtained from the individual(s) for the publication of any potentially identifiable images or data included in this article.

## Author contributions

AM: Conceptualization, Data curation, Formal analysis, Investigation, Methodology, Project administration, Visualization, Writing – original draft, Writing – review & editing. AS: Conceptualization, Data curation, Formal analysis, Investigation, Methodology, Project administration, Supervision, Validation, Visualization, Writing – original draft, Writing – review & editing. GS: Conceptualization, Data curation, Formal analysis, Investigation, Methodology, Software, Writing – original draft, Writing – review & editing. AD: Data curation, Formal analysis, Investigation, Writing – review & editing. RM: Data curation, Formal analysis, Investigation, Writing – review & editing. LF: Conceptualization, Data curation, Formal analysis, Investigation, Methodology, Validation, Writing – review & editing. AO: Data curation, Formal analysis, Investigation, Writing – review & editing. HM: Conceptualization, Formal analysis, Funding acquisition, Investigation, Methodology, Project administration, Supervision, Validation, Visualization, Writing – review & editing. RP-S: Conceptualization, Funding acquisition, Project administration, Supervision, Validation, Visualization, Writing – review & editing.
